# The modulatory role of internet-supported mindfulness-based cognitive therapy on extracellular vesicles and psychological distress in people who have had cancer: a protocol for a two-armed randomized controlled study

**DOI:** 10.1186/s13063-022-06045-x

**Published:** 2022-02-05

**Authors:** Diana R. Pereira, Eunice R. Silva, Carina Carvalho-Maia, Sara Monteiro-Reis, Catarina Lourenço, Rita Calisto, Ricardo João Teixeira, Linda E. Carlson, Genevieve Bart, Seppo J. Vainio, M. Goreti F. Sales, Carmen Jerónimo, Rui Henrique

**Affiliations:** 1 Psychology Service, Portuguese Oncology Institute of Porto (IPOP), Rua Dr. António Bernardino de Almeida, 4200-072 Porto, Portugal; 2 Cancer Genetics Group, Research Centre of Portuguese Oncology Institute of Porto (CI-IPOP), Rua Dr. António Bernardino de Almeida, 4200-072 Porto, Portugal; 3 RISE@CI-IPOP (Health Research Network), Portuguese Oncology Institute of Porto (IPOP), Rua Dr. António Bernardino de Almeida, 4200-072 Porto, Portugal; 4 Porto Comprehensive Cancer Centre (Porto.CCC), Rua Dr. António Bernardino de Almeida, 4200-072 Porto, Portugal; 5 Cancer Biology and Epigenetics Group, IPO Porto Research Center (GEBC CI-IPOP), Portuguese Oncology Institute of Porto (IPOP), Rua Dr. António Bernardino de Almeida, 4200-072 Porto, Portugal; 6 Cancer Epidemiology Group-Research Center, Portuguese Oncology Institute of Porto (IPOP), Rua Dr. António Bernardino de Almeida, 4200-072 Porto, Portugal; 7 Department of Epidemiology, Portuguese Oncology Institute of Porto (IPOP), Rua Dr. António Bernardino de Almeida, 4200-072 Porto, Portugal; 8 CINEICC, Faculty of Psychology and Educational Sciences, University of Coimbra, Rua do Colégio Novo, 3000-115 Coimbra, Portugal; 9 REACH, Mental Health Clinic, Rua de Camões, 218, 4° andar, sala 2, 4000-138 Porto, Portugal; 10 Department of Oncology, University of Calgary, Calgary, AB T2N 3C1 Canada; 11 Disease Networks Research Unit, Laboratory of Developmental Biology, Faculty of Biochemistry and Molecular Medicine, Infotech Oulu, Kvantum Institute, University of Oulu, 90570 Oulu, Finland; 12 BioMark@UC, Department of Chemical Engineering, Faculty of Sciences and Technology, University of Coimbra, Rua Sílvio Lima, 3030-790 Coimbra, Portugal; 13 BioMark Sensor Research/CEB, Centre of Biological Engineering of Minho University, Campus de Gualtar, 4710-057 Braga, Portugal; 14 Department of Pathology and Molecular Immunology, Institute of Biomedical Sciences Abel Salazar, University of Porto (ICBAS-UP), Rua de Jorge Viterbo Ferreira, 228, 4050-313 Porto, Portugal; 15 Department of Pathology, Portuguese Oncology Institute of Porto (IPOP), Rua Dr. António Bernardino de Almeida, 4200-072 Porto, Portugal

**Keywords:** Cancer, Distress, Extracellular vesicles, Internet, Online, Mindfulness-based cognitive therapy, Mindfulness-based intervention, Randomized controlled trial

## Abstract

**Background:**

Mindfulness-based interventions (MBIs) have been used in oncology contexts as a promising tool with numerous benefits for various health-related and psychosocial outcomes. Despite the increasing popularity of MBIs, few randomized controlled trials (RCTs) have examined their effects upon biological parameters. Specifically, no previous study has examined the effects of MBIs on extracellular vesicles (EVs), which are potentially important markers of health, disease, and stress. Moreover, the lack of RCTs is even more limited within the context of technology-mediated MBIs and long-term effects.

**Methods:**

The current study protocol presents a two-arm, parallel, randomized controlled study investigating the effects of internet-supported mindfulness-based cognitive therapy (MBCT) compared with treatment as usual (TAU). Primary outcomes are psychological distress and EV cargo of distressed participants with previous breast, colorectal, or prostate cancer diagnoses. Secondary outcomes are self-reported psychosocial and health-related measures, and additional biological markers. Outcomes will be assessed at baseline, 4 weeks after baseline (mid-point of the intervention), 8 weeks after baseline (immediately post-intervention), 24 weeks after baseline (after booster sessions), and 52 weeks after baseline. Our goal is to recruit at least 111 participants who have been diagnosed with breast, prostate, or colorectal cancer (cancer stage I to III), are between 18 and 65 years old, and have had primary cancer treatments completed between 3 months and 5 years ago. Half of the participants will be randomized to the TAU group, and the other half will participate in an 8-week online MBCT intervention with weekly group sessions via videoconference. The intervention also includes asynchronous homework, an online retreat after the fifth week, and 4 monthly booster sessions after completion of the 8-week programme.

**Discussion:**

This study will allow characterizing the effects of internet-based MBCT on psychosocial and biological indicators in the context of cancer. The effects on circulating EVs will also be investigated, as a possible neurobiological pathway underlying mind-body intervention effects.

**Trial registration:**

ClinicalTrials.govNCT04727593 (date of registration: 27 January 2021; date of record verification: 6 October 2021).

**Supplementary Information:**

The online version contains supplementary material available at 10.1186/s13063-022-06045-x.

## Introduction

Cancer remains one of the leading causes of death and accounted for 19.3 million new cases and 9.9 million deaths worldwide in 2020 [1, 2]. By 2040, these numbers are expected to increase to 30.3 million new cases and 16.3 million deaths [2]. However, survival rates are also increasing due to major advances in cancer diagnosis, treatment, and follow-up, which means that cancer will increasingly impact the lives of many people. This creates a need to control disease progression and recovery pathways to optimize the well-being, quality of life, and daily functioning of people with cancer.

Distress is among the main factors that negatively affect the quality of life of cancer patients, regardless of the type of cancer or the stage of disease progression [3–6]. It is defined as an unpleasant affective experience that affects psychological (cognitive/behavioural/emotional), physical, social, and/or spiritual domains of functioning. As a result, the ability to cope with the illness experience is severely impaired [7, 8]. Distress involves various experiences (e.g. anxiety; depression; fear; rumination) that vary from person to person and over time [5, 7]. Importantly, this is a common experience in people with cancer from the time of diagnosis through disease remission and long-term survivorship [4]. Hence, distress management becomes crucial but encounters numerous barriers, including underestimation of psychosocial needs and the stigma of receiving psychosocial support. Accordingly, many sufferers do not acknowledge their distress or avoid seeking professional help [8, 9].

Mindfulness-based interventions (MBIs) have been investigated as a viable approach to managing cancer distress and have become increasingly popular over the last two decades [10, 11]. These interventions are derived from ancient Buddhist meditation practices. They focus on bringing attention to the present moment, in an intentional, open, non-reactive, and non-judgmental way [12]. They aim to cultivate an attitude of observation, patience, and acceptance toward negative stressful experiences, rumination, and anxiety, rather than an attitude of judgment and avoidance [13].

In the context of cancer, the commonly used MBIs are mindfulness-based stress reduction (MBSR), mindfulness-based cognitive therapy (MBCT), and mindfulness-based cancer recovery (MBCR) [14]. These MBIs may include a variety of formal mindfulness meditation practices, such as body scan, sitting, and walking meditation, as well as informal practices that promote the integration of mindfulness strategies into daily routines, such as observing body sensations and bringing awareness into routine activities [15]. These interventions have shown promising results on several health-related and psychosocial dimensions, including reductions in distress, depression, anxiety, and fatigue, and through improvements in quality of life, sleep quality, posttraumatic growth, and mindfulness (e.g. [16, 17]; see [10, 11, 18–20] for reviews). A recent meta-analysis of 29 randomized clinical trials found small to medium size effects of MBIs on psychological distress, depression, anxiety, fatigue, sleep disturbances, fear of cancer recurrence and pain, both after post-intervention and at follow-up [10].

MBIs are usually conducted in a face-to-face mode. However, this presents difficulties and challenges, as face-to-face activities are not accessible to all cancer patients and survivors. Barriers to in-person participation in MBIs include living long distances from the health care facility, limited transportation or time, greater burden of the disease and/or cancer treatment (including pain and fatigue), some degree of disability and full family and work schedules [21–24].

The limitations of conducting a face-to-face intervention can be overcome by the internet- or application-based psychological activities [25–29]. These approaches have several advantages, including easier management of the specific needs of cancer patients, survivors, and caregivers; easier integration of practices into personal daily routines; reduction of costs for both patients and institutions; and expansion of the accessibility of psychosocial interventions to more people [21, 22, 30–34]. The great relevance of digital technologies has been highlighted in the current COVID-19 pandemic [33], as they play a central role in bringing together health care professionals and patients [35]. This is particularly important in more vulnerable groups, such as cancer patients and survivors [36, 37]. Overall, digital health interventions now present a unique opportunity to consolidate and strengthen psychological interventions, improve the response of health care providers in terms of individual needs, and reduce financial and human resources constraints.

However, the benefits of implementing MBIs using Internet-based tools are unclear [25]. In the specific case of online MBIs, including both healthy and clinical groups, the literature shows positive effects on depression, anxiety, well-being, and mindfulness skills [38, 39]. In relation to cancer patients, several studies suggest that technology-mediated MBIs are feasible and beneficial in improving various psychosocial and health-related outcomes [14]. These include psychological distress, stress symptoms, fatigue, anxiety, depression, mood disturbances, sleep quality, quality of life, spirituality, mindfulness skills, fear of cancer recurrence, and rumination [21, 33, 40–51]. Moreover, greater improvements in anxiety, depression, and posttraumatic growth [14] were reported for online MBIs (compared to control conditions). However, there are other reports of lack of improvement in some of these outcomes, including perceived stress [48, 52], depression, anxiety, fatigue [44, 45, 53], posttraumatic growth [49], rumination [48], physical health-related quality of life [21, 42, 44], pain, spirituality, mindfulness skills [45, 48, 53], and sleep-related measures [45, 52]. Thus, the literature information on conducting MBIs with internet-based instruments in cancer care is inconclusive. This may be due to the use of different methods in the reported studies (e.g. type and characteristics of the intervention; mode of delivery; selected outcomes; timing of assessment), and the high variability of sociodemographic and clinical characteristics of the observed groups [14, 48, 52]. This also shows that systematic studies targeting Internet-based MBIs are needed to understand how this valuable tool can be used to benefit all, especially in times of pandemic.

To date, few randomized controlled trials (RCTs) have been published for online MBIs in cancer care [14] and only a small part of these assessed the stability and progression of improvements over time [42, 52, 54]. A subset of these studies showed improvements in psychological distress, anxiety, rumination, and quality of life after intervention [42, 52, 54], while other studies reported no gains in depression [52], fear of cancer recurrence, and physical-related quality of life [42]. This conflicting data may be justified by the use of different self-reported psychosocial and physical outcomes to monitor the effectiveness of online MBIs.

In this regard, beneficial online MBIs should also have a positive effect on biochemical markers, as has been demonstrated with face-to-face interventions [55–58], which could help to extend the benefits of the procedure beyond self-report, and monitor the extent of those benefits. Previously reported data support that face-to-face MBIs modulate immune function [59], contribute to increased telomerase activity [60], and preserve telomere length [61]. These interventions also appear to reduce pro-inflammatory gene expression and inflammatory signalling, as well as salivary cortisol levels [17]. Reductions in pro-inflammatory cytokines, such as interleukin-6 (IL-6), tumour necrosis factor-alpha (TNF-α), and interferon gamma (IFN-γ) [62, 63] along with increases in anti-inflammatory cytokines including IL-4, were also reported [62], supporting that MBIs may help balance immune system activity [55, 57]. In another example involving individuals with prostate cancer, a combination of MBSR with diet affected prostate-specific antigen (PSA) levels [64]. Many other biochemical markers would be of relevance to this topic, but there is not a set of biomarkers that have been selected as preferential for this kind of study [58].

Research work on intercellular communication and signalling through extracellular vesicles (EVs) and their contents (e.g. DNA; mRNAs; microRNAs; proteins; lipids) is rapidly increasing. EVs are cell messengers of health, disease, and stress that circulate through body fluids, and contain important information about the current state of an organism [65–69]. Thus, EVs are now considered a promising tool for signalling a wide variety of biological events.

In addition, EVs are already used in cancer screening, diagnosis, and progression, regardless of the organ from which the EVs originate [70, 71]. It has been shown that the secretion of EVs by malignant cells is higher than that of non-malignant ones and that their content also differs in terms of nucleic acid and proteins [72, 73]. Moreover, EVs of malignant cells can influence their neighbouring cells and establish and develop a tumour microenvironment that promotes cancer growth, invasion, and metastasis [70, 71]. Overall, cell-to-cell communication through these vesicles plays a role in the biochemical processes associated with cancer progression and metastasis [70–73], opening an opportunity of investigating biomarker candidates with clinical relevance and developing innovative therapeutic targets in cancer [70, 71, 73].

Specifically, central nervous system (CNS) EVs are viewed with great enthusiasm, because they contribute to better understanding of brain functioning, improve therapeutic management of neurodegenerative and neuropsychiatric clinical conditions [66, 67, 74–76], and may help clarify mind-body interactions that remain unclear despite major advances in science and technology. Moreover, EVs cross the blood-brain barrier, implying that circulating CNS-EVs may contain valuable information about how mind activities may impact the body.

As far as we know, no study has yet addressed the impact of psychological interventions such as MBIs on circulating EVs. It is not known whether they are altered by such interventions, which is not surprising given that EV research is in its infancy. There are also technical challenges involved in isolating CNS-derived EVs, from biofluids such as plasma [71, 73, 74, 76], which are currently being addressed by the project MindGAP (funded by the European Commission).

Therefore, the primary aim of this study is to observe the effect of an internet-based MBCT intervention (*vs*. treatment as usual) on CNS-EV cargo (objective measure) and psychological distress (subjective measure), in a sample of distressed people with a history of breast, prostate, and colorectal cancer.

A secondary objective is to monitor the direct impact of online MBCT on the overall immunological response (objective measure). For this purpose, several biochemical markers in the blood will be evaluated, including the inflammatory response of interleukins IL-1, IL-6, IL-8, IL-10, IFN-γ, and TNF, and C-reactive protein. Other biomarkers that are related to cancer recovery after disease termination are also evaluated, including telomerase activity, antigens related to cancer (cancer antigen—CA 15-3; prostate-specific antigen—PSA; carcinoembryonic antigen—CEA), and other health-related markers (adrenocorticotropic hormone; erythrocytes; glycosylated haemoglobin). While biomarkers of cancer recovery are routinely monitored in recovered patients, biomarkers of immunological response are intended to provide a more general picture of the impact of MBCT upon the overall immunological response. This is important to generate relevant data to support any scientific conclusion about the mind-body interaction.

Similarly, secondary subjective psychosocial parameters are also considered, namely quality of life, fear of cancer recurrence, emotion suppression, mindfulness abilities, sleep quality, posttraumatic growth, health-related behaviours (physical activity; nicotine dependence), and perceived social support. Care was taken to select brief measures, to minimize participant burden.

To evaluate these global objectives, a one-site two-arm parallel randomized controlled superiority study with a 1:1 allocation ratio to internet-supported MBCR vs. TAU will be conducted, with outcomes measured at five distinct time points: (1) baseline; (2) 4 weeks after baseline (mid-point of the intervention, some self-report measures only); (3) 8 weeks after baseline (post-intervention); (4) 24 weeks after baseline; and (5) 52 weeks after baseline. The last two time points will provide evidence for evaluating the long-term effects of online MBCT.

## Methods

The Standard Protocol Items: Recommendations for Intervention Trials (SPIRIT) 2013 checklist that supports this study can be consulted in Additional file 1.

### Study setting

The study is conducted at the Instituto Português de Oncologia do Porto (IPOP; Portuguese Oncology Institute of Porto), a reference hospital for cancer in northern Portugal. Data are being collected from Portuguese patients of this hospital. Public data about this trial can be obtained on the website of the project funding this study [77]. The majority of the interactions with participants are being conducted via internet-based platforms.

### Participants

#### Eligibility

The inclusion criteria are summarized in Table 1. It is noteworthy that breast, prostate, and colorectal cancer diseases were selected, as they are the most common cancers in Portugal, with both sexes included [78]. Completion of primary treatments was also considered to minimize the impact of cancer treatment on biological markers, also considering that psychological distress may still be present in people who have had cancer, regardless of survival stage (at least up to 5 years) [79].

**Table 1:** Eligibility criteria

Inclusion	Exclusion
• Age between 18 and 65 years old* • Diagnosis of breast, prostate, or colorectal cancer (cancer stage I to III) • Primary cancer treatments completed between 3 months to 5 years previously (ongoing hormonal therapy will be included) • Experience of significant distress at the time of inclusion (DT ≥ 4) • Willingness to accept randomization to one of the two study conditions and participation in the intervention and data collection for the duration of the study • Ability to speak, read, and write in Portuguese and literacy to autonomously complete the self-report measure • Sufficient digital literacy and access to a device (e.g. smartphone; tablet; computer) with a camera, microphone, and internet	• Concurrent diagnosis of severe psychiatric condition(s) (e.g. bipolar disorder; psychosis; substance abuse; suicidal ideation) • Concurrent diagnosis of autoimmune disorder • Current use of antipsychotics • Current use of anti-inflammatory medication (e.g. corticotherapy) • Ongoing trastuzumab therapy • Participation in a structured mindfulness programme (e.g. MBCR; MBCT; MBSR) in the past 5 years • Currently attending psychological consultation • Being pregnant or breastfeeding

*Note*. *DT* distress thermometer, *MBCR* mindfulness-based cancer recovery, *MBCT* mindfulness-based cognitive therapy, *MBSR* mindfulness-based stress reduction. *This age criterion was considered due to other study of the MindGAP project in which healthy blood donors reporting low psychological distress will be compared with distressed participants diagnosed with cancer for a better characterization of the CNS-EVs. This characterization is important given that CNS-EVs are a novel approach in both healthy and clinical groups. As blood donors are aged between 18 and 65 years, the same criterion was applied to the participants diagnosed with cancer to have a closer age match between groups

In addition, participants must have significant distress at the time of inclusion, defined by a score of 4 or higher on the National Comprehensive Cancer Network (NCCN) Distress thermometer [8], as used in previous MBI studies [23], and considering that this cut-off score indicates moderate distress related to cancer [80, 81].

#### Recruitment

Two main strategies are planned to reach the target population. The first is based on obtaining a list of potential participants from the hospital’s Research Outcome Laboratory, filtered by relevant criteria, such as type of cancer and treatments. These potential participants will receive an informative email and/or SMS inviting them to visit the official website of the MindGAP project [77]. This website provides participants with more information about the study, including how to register. In the second variant, IPOP’s social networking sites (e.g. Facebook; Instagram; Institutional website) will be used to conduct regular dissemination and invite potential participants to visit the MindGAP’s website. Additionally, some health professionals working directly with the target groups will inform potential participants that the current study is recruiting and that more information can be obtained via the website.

In any case, participants can request more information by email or phone. Once contacted, participants receive a link to an online eligibility screening survey. This survey consists of an informed consent form and sociodemographic and health-related questions. If eligibility criteria are met, participants are invited to proceed in the study and to complete the baseline assessment. If any risk is identified (e.g. suicidal ideation), participants are informed about how to access specialist care services.

#### Informed consent

Informed consent is obtained directly from potential participants, via the online survey mentioned earlier. Specifically, participants are asked to confirm that they have read the information provided and are willing to collaborate, by checking a box. They will also be asked to authorize the research team to contact them regarding this study, access individual clinical records, and record the intervention sessions. Alternatively, participants will have the option to check a box to end their collaboration.

#### Sample size estimation

Because no previous study has examined circulating EVs in relation to MBIs and cancer, we could not use a specific effect size in estimating the sample size. Nonetheless, a recent systematic review examining the effects of Internet-based MBIs on psychological distress (anxiety and depression) found a median Cohen’s *d* value between 0.38 and 0.42 for the other primary outcome, psychological distress [14]. By using G*Power-3.1 statistical software [82] and considering repeated-measures ANOVA related to within-between interaction (group × time interaction), with an alpha significance level of 0.05 and an effect size of 0.38 (as calculated in [83]), a sample of 84 participants would allow for a power of 0.80. Additionally, previous studies showed dropout rates ranging from 12.1 to 32% for the intervention group and from 6 and 17.4% for the control group, when considering the period immediately following the intervention [23, 32, 40, 47, 49, 52, 84]. At follow-up, dropout rates were about 30.8% in the intervention group and 19.6% in the control group [52]. Considering the highest dropout rate (32%), 27 participants should be added to the estimated sample, resulting in a minimum of 111 participants to be included in the study.

### Intervention procedures

**Fig. 1:**
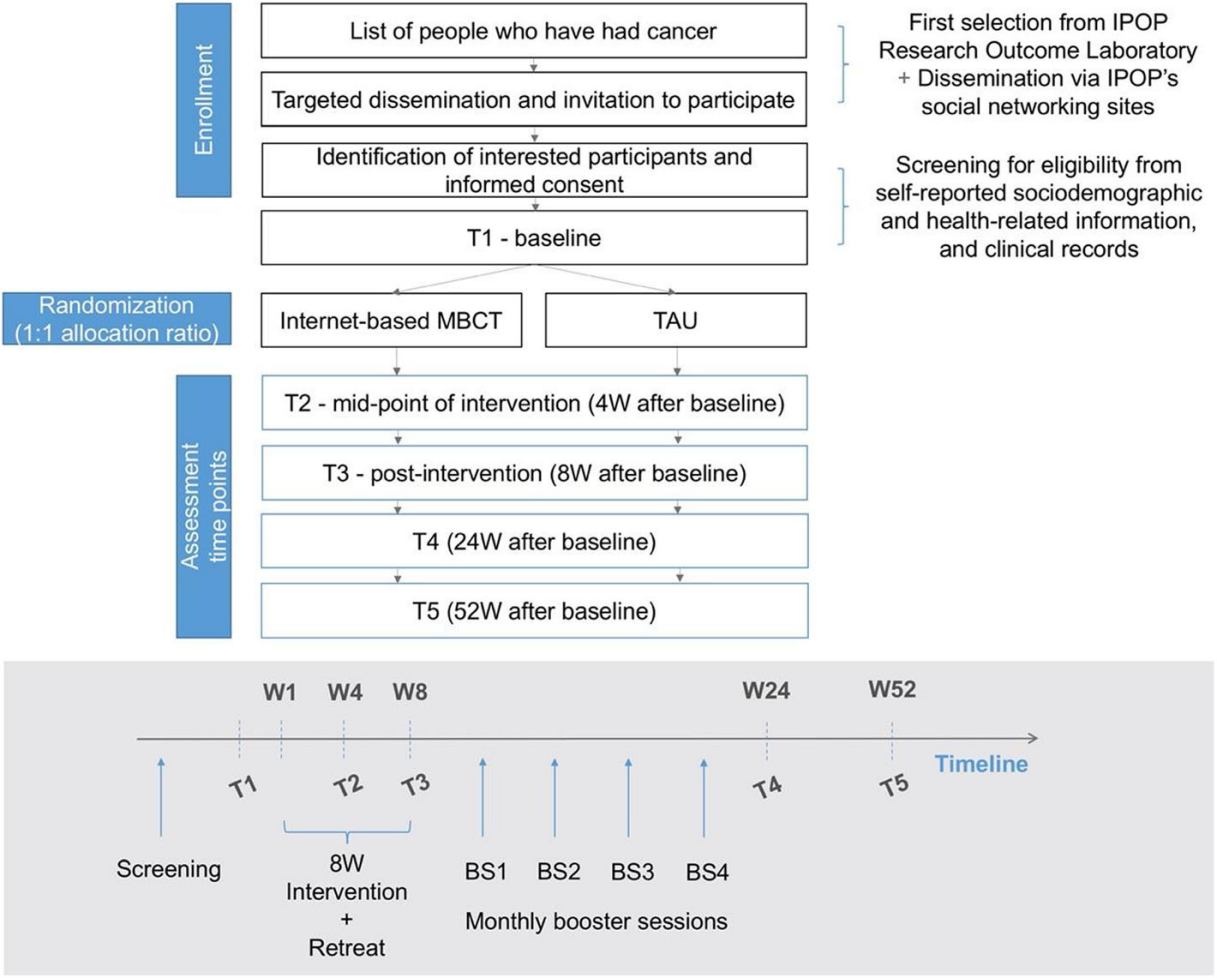
Study design and participant flow diagram (BS: booster session; IPOP: Instituto Português de Oncologia do Porto; MBCT: mindfulness-based cognitive therapy; TAU: treatment as usual; W: week)

A schematic representation of the study design from recruitment to the final follow-up time point is provided in Fig. 1. Briefly, after enrolling in the study, participants must complete the baseline survey, which consists of both biological and self-assessments. It is worthy to note that the self-reported measures are being completed via online surveys, while the biomarkers require the participants to visit the hospital for a blood sample collection. The blood samples will be preferably collected during routine clinic visits that occur in all recovered patients. After completing the baseline, participants will be randomized to one of the study conditions, internet-based MBCT or TAU. Subsequent study time points are at 4, 8, 24, and 52 weeks after baseline.

#### Experimental group

The MBCT programme [85] is a manualized group-based training programme that combines aspects of cognitive-behavioural therapy and aspects of the MBSR programme [86]. It was originally developed to reduce the risk of relapse and recurrence associated with major depression, but has also been used for other clinical conditions (e.g. cancer; chronic pain; vascular disease) with satisfactory results (see [87] for an overview). We selected this programme as it has been used in cancer patients with moderate to high levels of distress and has also shown promising results in an online setting [31, 41, 42, 84, 88, 89].

During the programme, participants learn mindfulness skills to bring attention to the present moment and foster increasing awareness of body sensations, thoughts, and feelings. This includes recognizing and accepting negative and unwanted thoughts, as well as the transitory quality of feelings. The idea is to allow the mind to move from automatic and spiralling patterns to a more conscious processing of mental and physical activities [90]. Overall, the awareness of the usual patterns of the self shall help to manage and accept unwanted thoughts and feelings.

The online MBCT programme that is being used in this study will be as close as possible to a face-to-face version. It consists of eight weekly 2-h sessions in a group setting (usually 12 participants), follow-up sessions, and an online retreat that addresses meditation skills and is led by a mindfulness facilitator [15, 85]. The general structure of the MBCT programme is as follows: the first four sessions are devoted to promoting basic mindfulness skills and psychoeducation regarding unhelpful thoughts; the last four sessions promote an accepting attitude towards unhelpful thoughts and feelings. Each session usually begins with mindfulness and meditation exercises, followed by group discussion, a review of homework activities, and the introduction to new exercises. A summary of the programme can be found in Table 2.

**Table 2:** Overview of the activities planned for the 8-week online MBCT group

Sessions (general theme)	Synchronous class activities	Asynchronous homework activities
Session 1 (recognition of the tendency to be on automatic pilot)	Establishing and reviewing ground rules Getting to know the group Mindfulness meditation exercises: eating a raisin mindfully; body scan; short breathing focus for 2–3 min	Body scan (audio-guided) Being mindful during a routine activity Mindful eating
Session 2 (promotion of awareness of how the mind responds to daily events and intensification of body focus)	Mindfulness meditation exercises: body scan; sitting meditation Identification of thoughts, feelings, and body reactions in response to a given daily event	Body scan (audio-guided) Mindful breathing Pleasant events calendar Being mindful during a different routine activity
Session 3 (introduction to breathing space as a way to focus on the present moment when dealing with the busy mind)	Mindfulness meditation exercises: sitting meditation; 3-min breathing space; mindful movement Calendar with unpleasant events	Mindful yoga (audio-guided) Stretch and breath exercises (audio-guided) Unpleasant events calendar 3-min breathing space
Session 4 (reinforcement of mindfulness as a way to stay in the present moment)	Mindfulness meditation exercises: sitting meditation; 3-min breathing space; mindful walking Completion of automatic thoughts questionnaire Discussion about MBCT based on a video material	Sitting meditation (audio-guided) Mindful yoga (audio-guided) 3-min breathing space 3-min breathing after acknowledging the appearance of unwelcome feelings and thoughts
Session 5 (promotion of a non-judgmental, open, and receptive attitude toward experience)	Mindfulness meditation exercises: sitting meditation; breathing space Preparation of the response plan when dealing with unwanted thoughts and feelings	Sitting meditation (audio-guided) 3-min breathing space 3-min breathing after acknowledging the appearance of unhelpful feelings and thoughts
Session 6 (unpleasant thoughts and feelings do not represent reality)	Mindfulness meditation exercises: sitting meditation; 3-min breathing space Exercise on thoughts, feelings, and considering different perspectives Discussion of breathing space as way to focus on the present before delving into a wider perspective on thoughts, feelings, and occurring events	Combination of previous audio-guided exercises 3-min breathing space 3-min breathing after acknowledging the appearance of unhelpful feelings and thoughts Continue to work on the response plan
Session 7 (acknowledge warning signs and plan preventive strategies to tackle the occurrence of unpleasant events)	Mindfulness meditation exercises: sitting meditation; 3-min breathing space; mindful walking Investigate associations between activity and mood Have a list of pleasant activities and how to schedule them Continue to work on the response plan in pairs and then extend it to the group	From the practiced exercises, generate a plan of practice to be incorporated into daily routines 3-min breathing space 3-min breathing after acknowledging the appearance of unhelpful feelings and thoughts Continue to work on the response plan
Session 8 (maintain mindfulness-related practices on a regular basis)	Mindfulness mediation exercises: body scan and concluding meditation Continue to work on the response plan and detection of early warning signs Review and personal reflections regarding the programme (feedback questionnaire) Discussion on how to keep daily practices	Keep on practicing

*Note*. *MBCT* mindfulness-based cognitive therapy

Synchronous sessions and interactions will be conducted via videoconference with the facilitator and other group members (maximum of 12 participants). In addition to the online sessions, participants will be encouraged to complete daily asynchronous homework assignments using support materials created and provided by the programme. For this purpose, participants will be provided with audio-guided exercises shared via an online file hosting service and mp3 players. Participants will be expected to complete daily practices of 10–40 min six times per week. Home practice will be monitored weekly using online surveys. A 4-h online retreat is proposed after the fifth week of intervention, and 4 monthly booster and consolidation sessions of 2 h are scheduled after the synchronous sessions.

The mindfulness instructor(s) responsible for the intervention will be mental health professionals with training and clinical experience in MBCT and following UK good practice guidelines from the mindfulness-based teacher trainer network [91]. A random pool of sessions from different phases of the programme will be transcribed and compared to the original protocol to assess the level of adherence to the intervention protocol by the therapist(s). For this purpose, sessions will be recorded with the consent of the participants and the mindfulness instructor(s). These recordings can be used to support supervision or intervention by the mindfulness instructor(s) [13].

#### Treatment as usual (TAU)

Participants in TAU group will follow the routine intervention protocol established by the hospital for needs assessment, referral, follow-up, and management of individuals with significant distress difficulties. Participants will be monitored for recurrence of cancer or other health problems, changes related with pharmacological and non-pharmacological interventions, and the occurrence of other major life events.

#### Criteria for discontinuing intervention

In the group receiving MBCT, along with the weekly daily practice survey, participants will be asked to rate their participation in the programme, from harmful to helpful. This will allow monitoring, identifying, and preventing unwanted effects associated to the intervention. If a negative effect is identified, the difficulties will be explored and could result in the intervention being discontinued for those participants. In addition, all participants have the right to terminate their participation at any time. Whenever possible, information about the main reason(s) for dropping out will be requested and recorded.

#### Adherence and feasibility

Implementing strategies to promote adherence and prevent dropout is of paramount importance, especially considering dropout rates ranging from 12.1 to 32% in intervention groups [23, 32, 40, 47, 49, 52, 84] and variability in adherence in online programmes ranging from 52 to 83% [14]. Such strategies are also relevant when considering adherence in daily practice. Although current data are heterogeneous regarding the frequency and timing of home practice in online MBIs [14], MBIs are estimated to have an adherence rate of approximately 60% [92, 93]. In addition, studies reported that participants completed between 2 to 4 home exercises per week for online MBIs [46, 94]. Other studies documented reasons underlying for dropping out of online MBIs [21, 40, 52], including technical problems, insufficient technology-related skills, perceived high intensity of the programme, time commitment, lack of motivation, and cancer recurrence or other physical problems.

To avoid technological issues, efforts will be made to provide participants with information on how to complete the online surveys; what is required and how to participate in the videoconference sessions; steps to request specific assistance; in addition to answers to other questions that arise during the study that will be compiled and delivered to participants. Written instructions with step-by-step help information and links to online tutorials will be provided. In addition, interested participants may test the videoconference programme, equipment, and internet in a short session prior to the start of the intervention programme.

Issues such as the intensity and time commitment required by the programme and the compatibility of the programme with daily activities have been identified in the literature as significant barriers to participation [24, 34]. Therefore, flexibility in daily practice is expected. Previous findings suggested that participants prefer short, 10-min mindfulness meditations and more frequently employ body scan exercises, followed by formal sitting meditations, loving-kindness meditations, mindful yoga, and silent meditations [47]. This highlights the need to adapt home practice to a daily routine and offer different exercises with varying lengths to easily accommodate individual preferences [44, 47, 51].

Motivation during the programme is supported and intensified in synchronous group sessions led by instructors, as interpersonal interactions and peer group support have been identified as relevant aspects in online MBIs [21, 31, 34, 53]. Previous studies indicated that participants need to receive reminders about daily training [44]. MBIs that include reminders have been shown to be more likely to have greater effects on outcome measures than MBIs without reminders [28]. Reminders may also lead to better completion rates [95]. However, there is a lack of evidence in the literature about how often these reminders should be given to promote engagement and not an opposite effect (see [14] for a discussion). In this study, we will adopt some of the strategies reported in previous studies, including twice-weekly email reminders [47], reinforced reminders during the synchronous sessions [96], and additional reminders to unresponsive participants [22, 23]. In addition to these interventions, four booster sessions will be implemented to avoid the feelings of disengagement that participants may experience after weekly sessions [97].

The following feasibility measures are considered: recruitment rates (e.g. the proportion of individuals who responded with interest to our invitation; the proportion of individuals who agreed to participate in the study, regardless of eligibility; the proportion of individuals who completed the first assessment point—T1—and began the intervention); adherence to practice (e.g. number of attended sessions; the number of homework assignments completed per week; the average time spent practicing at home each day); programme completion and dropout rates; and programme satisfaction.

Programme satisfaction will be monitored through several approaches: (1) at the third data collection point, participants will receive an online written semi-structured questionnaire; (2) mindfulness instructor(s) will be invited to participate in a focus group with the research team; (3) a subset of participants will be invited to complete an online adaptation of the Client Change Interview (CCI; [98]; European Portuguese adaptation by [99]). The CCI qualitatively assesses how participants experienced the intervention, perceived changes during the intervention, what these changes are due to, positive and negative aspects related to the intervention, and suggestions. In this interview, participants use three 5-point Likert-type scales to rate: expected change (1—very much expected; 5—very much surprised by it); change without intervention (1—clearly would not have happened; 5—clearly would have happened anyway); the value of the change (1—not at all important; 5—extremely important).

### Outcome measures

The primary and secondary outcomes collected via self-report measures are briefly described and summarized in Table 3. In short, primary outcomes are CNS-EV cargo (objective measure) and psychological distress (self-report measure). Secondary outcomes include self-reported psychosocial and health-related measures (quality of life; fear of cancer recurrence; emotion suppression; mindfulness abilities; sleep quality; posttraumatic growth; health-related behaviours; perceived social support) and a variety of biomarkers (IL-1; IL-6; IL-8; IL-10; IFN-γ; TNF; C-reactive protein; CA 15-3; PSA; CEA; adrenocorticotropic hormone; erythrocytes; glycosylated haemoglobin).

**Table 3:** Summary of self-report primary and secondary outcomes, respective assessment measures, and screening measures

Outcome	Measure	Brief description
Primary		
Psychological distress	Depression Anxiety Stress Scales-21 (DASS-21 [100]; EP version by [101])	It is a public domain instrument that evaluates negative affective states, being one of the available measures to assess psychological distress [102, 103]. It is also used in RCTs of manualized MBIs in the oncological context [104]. It is an abbreviated version of the original version of 42 items and can be administered to people aged 18 or more. It comprises 21 items, 7 measuring depression, 7 measuring anxiety, and 7 measuring stress. Specifically, participants are required to rate each item on a 4-point Likert-type scale as reference to how they have been feeling during the last week. The rating scale varied between 0 and 3, in which 0 represents “did not apply to me at all” and 3 represents “applied to me very much or most of the time”. The score for each subscale is obtained by summing the respective 7 items, whose result range between 0 and 21. In this study, a total score will be used by summing all items, with higher scores being indicative of higher self-reported negative affective experiences. The EP version yielded satisfactory internal consistency and convergent and discriminant validity [101]).
Secondary		
Emotion suppression	Expressive Suppression scale of the Emotion Regulation Questionnaire (ERQ [105]; EP version by [106])	It evaluates strategies of emotional regulation, particularly cognitive reappraisal, and expressive suppression. It is a brief questionnaire including 10 items, 4 dedicated to expressive suppression and 6 to cognitive reappraisal, contributing to the 2-factor structure. Each item is rated on a 7-point Likert-type scale, wherein 1 represents strongly disagree and 7 strongly agree. Two scores are derived, one related to cognitive reappraisal (ranging from 6 to 42) and the other related to expressive suppression (ranging from 4 to 28). Higher scores indicate higher employment of the regulation strategy under evaluation. The original study demonstrated that ERQ has good psychometric properties of internal consistency, test-retest reliability, convergent, and discriminant validity. These good properties have been replicated in different samples such as community samples (e.g. [107]) and people diagnosed with cancer, including in the Portuguese context (e.g. [108]). In the current study, only the expressive suppression scale will be used as an outcome.
Fear of cancer recurrence	7-item Fear of Cancer Recurrence Questionnaire (FCR7 [109])	It is a unidimensional screening measure of FCR to be used in oncological contexts. It is composed of 7 items, most of them rated in a 5-point Likert-type scale ranging from 1 (not at all) to 5 (all the time) and one item rated in a 10-point scale ranging from 0 (not at all) to 10 (a great deal). Higher scores are indicative of higher reported levels of FCR. As a reference, a score of 17 corresponds to a moderate level of FCR and a score of 27 corresponds to a high level. Satisfactory psychometric properties have been documented in terms of internal consistency, test-retest reliability (1-month), content and convergent validity [110]. As FCR7 is a recent questionnaire, no EP version is yet available. Thus, the research version is being developed in the context of this study.
Mindfulness	Five-Facet Mindfulness Questionnaire (FFMQ [111, 112]; EP version by [113])	It was developed based on a comprehensive analysis of different mindfulness questionnaires, supporting the notion that mindfulness is a multifaceted construct. Specifically, this questionnaire assesses mindfulness and self-awareness states in everyday life, incorporating five facets/subscales: observing, describing, acting with awareness, nonreactivity to inner experience, and nonjudging of inner experience. It encompasses 39 items, each one rated on a 5-point Likert-type scale ranging from 1 (never or very rarely true) and 5 (very often or always true). A score is derived for each facet. This questionnaire has been widely used in intervention studies. In addition, it has been adapted to different cultures with good results [114]. In the case of the EP version, satisfactory psychometric properties for the five-dimension solution in terms of internal consistency, convergent and discriminant validity had been reported [113].
Nicotine dependence	Fagerström Test for Nicotine Dependence (FTND [115]; EP version by [116])	It is a widely used questionnaire to assess nicotine dependence. It is a brief measure composed of 6 items. Two of the items are scored between 0 and 3, and the remaining are scored between 0 and 1. Thus, the total score ranges between 0 and 10. The EP version has been tested for internal consistency and test-retest reliability with satisfactory results. Also, a two-factor structure emerged, one related to cigarette consumption and the other related to morning smoking [116]. Of note, to complement the data regarding the consumption of substances that may interfere with the biological markers under study, questions concerning the weekly consumption of alcohol, coffee, coffee-based beverages, and tea, as well as the type of diet and its self-perceived quality will be added.
Physical activity	International Physical Activity Questionnaire – Short Form (IPAQ – SF; [117])	It evaluates physical activity and sedentary behaviour (sitting). In this study, the short form will be used because it is faster and easier to administer while maintaining similar reliability and validity properties as compared to the extended version. It contains 9 items encompassing the frequency (days) and time spent on walking, moderate-intensity activities, vigorous-intensity activities, and sedentary activities. The reference period to be used in the current study will be the “usual week”. An estimation of time (minutes) per week dedicated to each type of intensity (vigorous, moderate, walking) and sitting can be obtained. From these data it is possible to estimate the total weekly physical activity in MET minutes per week. Also, it is possible to categorize the people into 3 possible levels of physical activity: low, moderate, or high (the following protocol can be used: https://sites.google.com/site/theipaq/scoring-protocol). Concerning the psychometric properties, acceptable results were described in the study of Craig and collaborators [117], considering test-retest reliability (around 1 week), criterion (based on accelerometer), and concurrent (based on short and long versions) validity. Although scarce, the evidence available for the EP adaption is also reasonable [118].
Posttraumatic growth	Posttraumatic Growth Inventory (PTGI [119]; EP version by [120])	It is a self-report questionnaire that assesses positive psychological change in people that faced traumatic experiences. It is composed of 21 items structured in a 5-factor model: new possibilities (5 items); relating to others (7 items); personal strength (4 items); spiritual change (2 items); appreciation of life (3 items). Each item is rated on 6-point Likert-type scale ranging from 0 (“I did not experience this change as a result of my crisis”) to 5 (“I experienced this change to a very great degree as a result of my crisis”). The total score varies between 0 and 105, with higher scores depicting a greater degree of posttraumatic growth. The original study revealed satisfactory internal consistency, test-retest reliability (2 months), construct, concurrent, and discriminant validity. The Portuguese adaptation including participants diagnosed with breast cancer and non-clinical participants yielded reasonable psychometric properties [120, 121].
Quality of life	World Health Organization Quality of Life – Bref (WHOQOL-Bref [122, 123]; EP version by [124])	It is the abbreviated version of the 100-item instrument (WHOQOL-100), suitable for epidemiological and clinical trials, which evaluates the quality of life following the WHO’s proposal (i.e. the perception of an individual regarding one’s position in life given the cultural and social environment, as well as expectations, preoccupations, and goals). It includes 26 items arranged in one general facet and four specific domains: physical health, psychological, social relationships, and environment. Each item is rated having the last 2 weeks as the time period of reference and using a 5-point Likert-type scale. The scoring procedures can be consulted here: https://www.who.int/mental_health/media/en/76.pdf. Higher scores indicate higher self-reported quality of life. The original studies showed satisfactory psychometric properties in terms of internal consistency, test-retest reliability, construct, discriminant, and criterion validity. Similarly, satisfactory psychometric properties of validity and reliability were documented for the Portuguese adaptation [124], being successfully used in several studies with Portuguese oncological groups (e.g. [125, 126]).
Sleep quality	Basic Scale on Insomnia complaints and Quality of Sleep (BaSIQS [127])	It is a brief easy to administer self-report questionnaire that evaluates sleep quality and difficulties related to fall asleep and to maintaining sleep, considering a typical week in the last month. It encompasses 7 items rated on a 5-point Likert-type scale, and each one scored between 0 and 4. The total score ranges between 0 and 28, with the highest values being indicative of poor sleep quality. This questionnaire was initially developed and tested with groups of Portuguese higher education students, yielding good internal consistency, test-retest reliability, and convergent validity. Normative scores for males and females were presented based on percentiles, 25, 50, and 75. These good psychometric properties were also extended to a Portuguese community sample, and BaSIQS was able to distinguish between people with clinical insomnia and people with sleep disturbance [128]. In the current study, additional questions available in the plus version of BaSIQS will be included, namely the number of hours usually slept per night during week and weekend, frequency of nights per week wherein the participant is able to sleep the number of needed hours, frequency and duration of naps, and perceived subjective sleep problems.
Social support	Social Support Satisfaction Scale (SSSS [129])	It is a self-report questionnaire that assesses perceived social support. It is composed of 15 items distributed in four factors: satisfaction with friendship (5 items), intimacy (4 items), satisfaction with family (3 items), and social activities (3 items). Each item is rated on a 5-point Likert-type scale, ranging from totally agree to totally disagree, and scored between 1 and 5. A score can be obtained for each subscale, but the total score will be considered in this study. The total score can be extracted by adding each subscale score, and it ranges between 15 and 75, with higher scores representing higher perceived social support. The original study conducted with a Portuguese sample revealed good internal consistency, discriminant, and concurrent validity. Reasonable psychometric properties of validity and reliability were also reported study with a Portuguese and Brazilian sample of university students [130].
Screening		
Distress	Distress thermometer (DT [7])	It is a simple visual analogue scale with 11 points (from 0—no distress to 10—extreme distress) shaped like a thermometer and devised to screen the experience of distress during the last week in oncology populations. The higher the value selected, the higher the level of distress reported by the participant.
Global distress and mental health difficulties	Clinical Outcome Routine Evaluation – Outcome Measure (CORE-OM [131–133]; EP version by [134])	It is a self-report measure that incorporates 34 items organized in four different dimensions: well-being (4 items), symptoms (12 items), social and personal functioning (12 items), and risk behaviours (6 items). Each item is evaluated using a 5-point Likert-type scale that varies from 0 (not at all) to 4 (most or all the time). The time window of reference is the last week, and it takes about 5–10 min to complete. The original total score is obtained by averaging all the items. The mean score can be multiplied by 10 to facilitate interpretation, resulting in scores ranging between 0 and 40. Higher values denote a more severe level of global distress (see https://www.coresystemtrust.org.uk/instruments/core-om-information/). CORE-OM has been widely tested and demonstrates satisfactory concurrent and convergent validity, internal consistency, and test-retest reliability (1-week interval; [131–133]). Good internal consistency was also found in the EP adaption [134].
Sociodemographic and health-related information		It consists in information to be obtained directly from participants or clinical records: age; gender; years of formal education; nationality(ies); mother language(s); marital status; current regional location of residence; professional occupation and current occupational situation; average household income (mensal); children (number and ages); informal care provided to other persons; people living in the household; clinical history; familial cancer history, date and age of the cancer diagnosis, who detected the cancer, type of cancer and site, stage, cancer treatments (including start and end date); comorbidities (e.g. hypertension; diabetes mellitus; autoimmune diseases such as lupus, thyroiditis, rheumatoid arthritis); history of surgical interventions; pharmacological treatments and possible adjustments in the last 3 months; psychological intervention; psychiatric intervention; mindfulness meditative practices; menopause signs and symptoms in the case of female participants.

*Note*. *BaSIQS* Basic Scale on Insomnia complaints and Quality of Sleep, *CORE-OM* Clinical Outcome Routine Evaluation - Outcome Measure, *DASS-21* Depression, Anxiety Stress Scales-21, *DSM* Diagnostic and Statistical Manual, *DT* distress thermometer, *EP* European Portuguese, *ERQ* Emotion Regulation Questionnaire, *EVs* extracellular vesicles, *FCR-7* 7-item Fear of Cancer Recurrence, *FFMQ* Five-Facet Mindfulness Questionnaire, *FTND* Fagerström Test for Nicotine Dependence, *ICD* International Classification of Diseases, *IPAQ-SF* International Physical Activity Questionnaire - Short Form, *MBIs* mindfulness-based interventions, *n/a* not applicable, *PTGI* Posttraumatic Growth Inventory, *RCT* randomized controlled trial, *SSSS* Satisfaction with Social Support Scale, *WHOQOL-Bref* World Health Organization Quality of Life – Bref

The timing of these measures is shown in Table 4. All psychosocial measures employed at baseline (T1) are used at T3, T4, and T5. In the fourth week after the baseline (the fourth session in the case of the intervention group), self-reported measures of distress and mindfulness will be used. This intermediate point (T2) was considered as previous studies have suggested that four sessions are the minimum satisfactory intervention dose [14, 52, 84, 89]. Few measures were selected at T2 to avoid participant burden. Regarding biological samples, the plans are to collect them at T1, T3, and T4. Of note, participants will receive financial compensation for the costs they incur traveling to IPOP to participate in the blood collections.

**Table 4:**
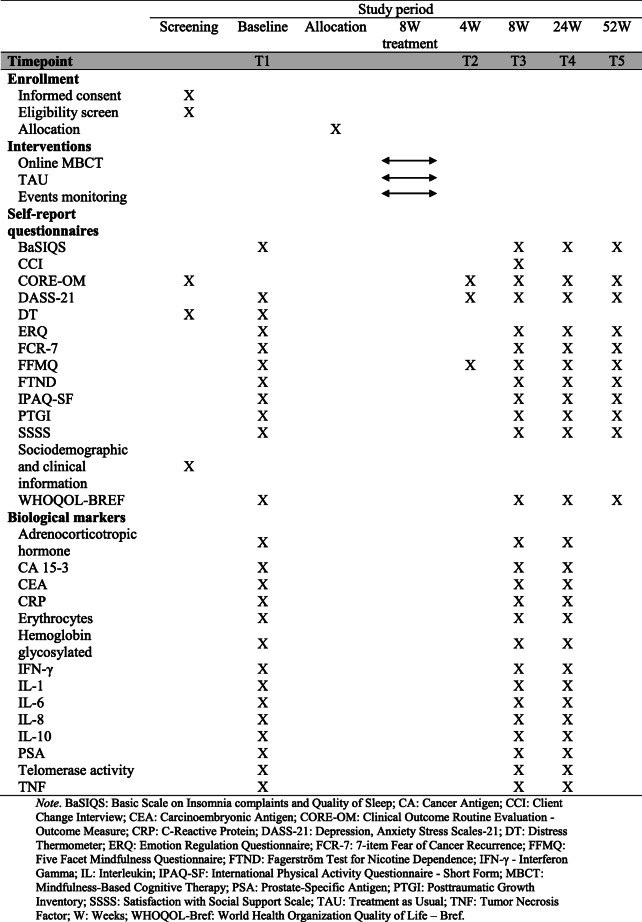
Schedule of enrolment, interventions, and assessments

*Note*. *BaSIQS* Basic Scale on Insomnia complaints and Quality of Sleep, *CA* cancer antigen, *CCI* Client Change Interview, *CEA* carcinoembryonic antigen, *CORE-OM* Clinical Outcome Routine Evaluation - Outcome Measure, *CRP* C-reactive protein, *DASS-21* Depression, Anxiety Stress Scales-21, *DT* distress thermometer, *ERQ* Emotion Regulation Questionnaire, *FCR-7* 7-item Fear of Cancer Recurrence, *FFMQ* Five-Facet Mindfulness Questionnaire, *FTND* Fagerström Test for Nicotine Dependence, *IFN-γ* interferon gamma, *IL* interleukin, *IPAQ-SF* International Physical Activity Questionnaire - Short Form, *MBCT* mindfulness-based cognitive therapy, *PSA* prostate-specific antigen, *PTGI* Posttraumatic Growth Inventory, *SSSS* Satisfaction with Social Support Scale, *TAU* treatment as usual, *TNF* tumour necrosis factor, *W* weeks, *WHOQOL-Bref* World Health Organization Quality of Life – Bref

For each self-report measure, the total score obtained for scale and/or subscales will be used as outcomes. Measures examining the eligibility criteria and the sociodemographic and health-related information are also reported in Table 3.

To determine EVs cargo, total EVs will be isolated from platelet-free plasma samples by ultracentrifugation, followed by a purification step for CNS-derived EVs by immunoprecipitation with CNS-specific antibodies. Since we are interested in CNS-derived EVs, brain-related microRNAs will be measured to verify the origin of the EVs. This is achieved by isothermal nucleic acid amplification method. Results are measured by luminescence.

To evaluate the biochemical markers, inflammatory response biomarkers (IL-1, IL-6, IL-8, IL-10, IFN-γ, TNF) will be detected in serum by multiplex immunoassay (MILLIPLEX® Multiplex Assays Using Luminex®). Similarly, various cancer antigens (CA 15-3 and CA 19.9 in units/mL, PSA in ng/mL, CEA in ng/mL) will also be detected by immunoassay to monitor cancer recurrence. Other health-related biomarkers will include c-reactive protein (mg/L), telomerase activity, erythrocyte number (million/mm^3^), adrenocorticotropic hormone (pg/mL via immunoassay analyser), and glycosylated haemoglobin (mmol/mol via high-performance liquid chromatography).

### Assignment of intervention

#### Allocation

Participants will be randomly assigned to one of the two arms: MBCT or TAU groups (1:1 allocation ratio). Randomization blocks will be created using informatic tools and the entire process will be overseen by an investigator who will not be directly involved in the intervention or assessments. This investigator will inform the research team of the final allocation of each participant.

#### Blinding

Prior to randomization, individuals involved in the study (participants, study coordinators, mindfulness instructor(s), research assistants, statistician) will be blinded to the study conditions. After randomization, the research team will inform participants about group membership. Similar to previous studies in the field [14], and given the specificities and resources available in the current study, it is impossible to guarantee further blinding of participants and staff irrespective of their role. This unblinding also applies to the data analysts as most of the team members involved in the data collection will participate in the data management and analysis. Even so, the team members not collaborating directly in the data collection, such as the team dedicated to the characterization of the CNS-derived EVs, will be blinded to the study conditions where possible.

### Data collection and management

After registration, participants will be assigned randomly generated identification codes. They will be asked to keep and use the code to avoid inserting sensitive personal data during their participation. Correspondence between the code and participant’s personal information (e.g. name; contact information; clinical identification in IPOP) is stored in a metadata file that is encrypted with a password and accessible only through the IPOP’s computer, which is protected by institutional security measures.

Data on sociodemographic and health self-report, screening, and primary and secondary outcome measures will be stored in the online survey service used here, LimeSurvey, which adheres to user privacy policies. Data will be periodically copied to an IPOP computer, where they are processed and inserted into excel files. These files will only be accessible to members of the IPOP research team via passwords or physical keys to open specific doors in the case of paper-based documentation, which will be kept in cabinets and locked.

Biological marker data will be obtained from blood samples collected from participants using a 21- to 23-gauge needle into 3 EDTA tubes (≤ 12 mL) and 1 nonadditive tube (4 mL). The standard tubes are stored at 4 °C for a maximum of 180 min. Tubes will be centrifuged at 2500*g* for 30 min at 4 °C. The upper third of plasma will be transferred to sterilized 2 mL tubes, and the plasma samples will be aliquoted and stored at − 80 °C in the hospital biobank until further use. The tubes will be labelled with a reference to the MindGAP project and a participant identification code to ensure anonymization during the blood collection and processing. Prior to EV isolation, a centrifugation step at 5000*g* for 15 min is performed in thawed plasma samples, to remove most of the remaining platelets.

For both self-reported and biological data, a privacy impact assessment has already been submitted, analysed, and approved by the IPOP’s Data Protection Officer.

### Statistical methods

Screening data for errors and missing values is the first step in the data analysis plan. Missing data will be managed and avoided during the data collection by using mandatory fields in the online surveys and retrieving the data from participants when possible. Data from participants who completed fewer than 4-weekly online MBCT sessions will not be considered further, as four sessions were set as the minimum number required for participation [14].

For each group (MBCT and TAU), a summary of baseline sociodemographic and health data will be provided, including number and percentages, mean, standard deviation, median, first and third quartiles, minimum and maximum. Using the sociodemographic and health data, as well as the primary and secondary measures, comparisons will be made between groups to examine whether there are differences between groups at baseline. Similarly, differences at baseline between participants who will complete the study and those who will drop out will be examined. The presence of differences in variables may warrant the need to examine and/or adjust for their influence on outcome changes in the primary analyses. This summary also presents the within-group effect sizes (Cohen’s *d*—equation 11.10, from [135]) for each outcome across time (i.e. T1 to T2; T1 to T3; T1 to T4; T1 to T5).

The total scores of each primary and secondary measure will be used as outcomes in multiple linear mixed models (LMMs). Each model will include the interaction between group and time, while participants will be treated as a random effect. The visual inspection of Q-Q plots will be conducted to check the normal distribution of residuals. Examination of factors such as sex, type of cancer, and cancer stage will be approached by running separate models for each factor. LMM analyses can be employed using, for instance, lmer4 [136] and lmerTest [137] in R software [138].

Descriptive statistics for feasibility indicators (see “Adherence and feasibility” section) will be documented. In the case of programme satisfaction, a qualitative analysis of the information collected in the semi-structured interview, focus groups, and CCI will be conducted [98]. For this purpose, a computer-assisted thematic analysis will be used, following Braun and Clarke’s 6-step approach [139] (see also [140, 141]): (i) familiarization with the data (transcribing the non-written data to a written format and carefully reading all the material several times to become familiar with it); (ii) initiation of the coding process (identifying emergent units of meaning—codes—that are relevant to the research and can be used to tag similar data); (iii) generation of potential and broader themes (codes can be categorized according to the themes and a thematic map that contains associations between themes, including major and sub-themes, and between codes); (iv) review the themes (themes are reviewed to verify if they fit the coded data so that they can be deleted, modified, merged and/or separated; also, the accuracy of the thematic map is reviewed); (v) description of themes (provide a name, characterization, and a scope, and refine the themes step by step); (vi) report of analysis (write and describe in detail the process of analysis using data supporting the themes and discuss the results in light of the literature and research goals). As recommended, steps 1–3 will be conducted by at least two independent researchers and later brought into the group discussion to promote thematic and code reliability [142].

### Oversight and monitoring

No Data Monitoring Committee was designated given the nature of the study, i.e. psychosocial intervention with low risk. Nonetheless, participants in the intervention group will be monitored weekly for their experience in the intervention sessions. The aim is to detect adverse events associated with the intervention (see [14, 24] for some examples), to discontinue the intervention or, if necessary, to make a referral to specialist care services. The IPOP’s research team will be responsible for this monitoring process, and any incident will be documented in the participant flow diagram.

This study has no designated coordinating centre or trial steering committee. The IPOP’s research team have the responsibility to conduct and monitor the day-by-day tasks necessary to run the study. This team meets periodically to assess the conduct and progress of this study and to ensure compliance with the study protocol. There are also meetings every 3 months including the IPOP’s team members responsible for the study coordination (ERS, CJ, and RH) to discuss management and financial issues related to the study. The person responsible for the financial management of the study also participates in some of these meetings. Additionally, the study is discussed in the bi-annual consortium meetings of the MindGAP project [77]. This allows to share with the other research partners the current status of the clinical study, achievements and goals, being an opportunity to receive feedback and guidance. Other than these meetings, no formal auditing is planned as the IPOP has no audit department dedicated to this type of study.

Changes to the study protocol will first be submitted to the IPOP Ethics Committee and after approved updated accordingly in clinicaltrials.gov. The updates will also be reported to the MindGAP project research partners during the consortium meetings.

### Dissemination plans

The activities and results of the MindGAP project are updated on the project website [77] and disseminated on the project’s social networking platforms (Facebook, Twitter, LinkedIn, YouTube), which are linked on the project homepage. The public can interact with the research team through the former channels. The results of the study will be presented in scientific meetings and published in open-access journals. By the end of this study, it is planned to present the results to participants, healthcare professionals, and the community in an open session organized at IPOP. Social and traditional media platforms could also be used to reach a wider audience.

## Discussion

The motivation of this study is twofold: (1) to test the influence of MBCT practices on circulating CNS-derived EVs, investigated here for the first time; (2) and to further substantiate the impact of Internet-based MBCT on psychosocial and biological markers in an oncological context.

The use of CNS-derived EVs as tool for discovering biomarkers to support the psychosocial and biological changes that occur in the body following MBCT practices is a novel perspective never explored in the literature. The novel hypothesis presented here suggests that EVs may be involved in an important neurobiological mechanism of action underlying mindfulness interventions. Circulating CNS-EVs carry information to peripheral cells, and this information acts as instructions to these cells sent via mind-controlled events. In this hypothesis, EVs could lead to a further understanding of how mind-controlled activities can cause biological changes in the body.

There are challenges and limitations with this study that need to be acknowledged. First, the inclusion of participants with some level of digital literacy and access to digital devices and the Internet likely reflects sociodemographic characteristics, with younger and more educated participants being more mobilized [143]. Access to and use of information and communication technologies in Portugal is still closely associated with age and education, meaning that older and less-educated individuals are not as involved as other sociodemographic groups [144]. Accordingly, the results of this study may not apply to people with other sociodemographic characteristics. Nevertheless, this is an important step towards the development of digital mental healthcare services, whose underdevelopment in Portugal has been exposed by the current pandemic situation [145].

Moreover, despite the inclusion of specific types of cancer, which allows the formation of more homogeneous groups, as recommended in previous literature [14, 57], some individual characteristics and needs may be unconsidered. Accordingly, future studies could benefit from investigating other cancer types and stages (in treatment and palliative conditions [79]), exploring new ways to consider individual aspects [146], and including other outcomes (e.g. cognitive dysfunction, fatigue, pain) and possible associations with biological parameters [56].

Furthermore, given the novelty behind this study, the sample size of the objective primary outcome could not be adequately estimated for EV studies. Therefore, the current study may be underpowered to detect the effects of internet-based MBCT on circulating EVs. This problem could be compounded by difficulties with adherence and retention in this study, given the time and other specific demands of mindfulness practice [33]. With this concern in mind, specific strategies to mitigate dropouts were identified. Also, the qualitative methods proposed in this study might be useful in this regard.

The control data is obtained here by following TAU. This control condition was selected considering the available resources, the time window for conducting this clinical study, the previous use in online MBI studies [46, 48, 52, 53, 84, 89], and the desire for a year of follow-up comparison data (hence precluding a short waitlist period for the control group). We are aware that this approach does not allow control of specific (e.g. psychoeducational aspects) and nonspecific effects (e.g. group interactions and support; closer supervision; interaction with mental health professionals) [90]. An active control or even a comparison with another established intervention would be preferable, if possible [13, 90]. However, passive control conditions have been the most common in digital MBI research to date. As the field evolves, active control conditions are likely to become more common, as suggested by studies comparing face-to-face MBIs with Internet-based MBIs [31, 41, 42, 54, 84, 89] or studies using psychoeducational intervention as a comparison [32, 40].

Considering the challenges and limitations discussed earlier, it is important to say that not all scenarios are predictable. Therefore, the possibility of overcoming the challenges by adopting new or adapted strategies that may prevent the difficulties encountered throughout this study is anticipated, in a manner that provides valuable insights into health-related and psychosocial gains fostered by internet-based MBCT in an oncology context.

Overall, it is expected to confirm and clarify the role of CNS-EVs in altering the biochemical operation of peripheral cells in response to mind-related activities, while monitoring the extent of these changes in a wide range of subjective and objective parameters. This mechanism may open unprecedented opportunities to understand how the mind may interact with the body, in what quantitative dimension, and also provide tools for a mind-controlled activity that may benefit health.

## Trial status

The unique protocol ID is IPO/PI134b and the ClinicalTrials.gov Identifier is NCT04727593 (date of registration: 27 January 2021; date of record verification: 6 October 2021; https://clinicaltrials.gov/ct2/show/NCT04727593). The first wave of recruitment began on February 2021 and it is expected to continue until October 2022. The anticipated study completion date is March 1, 2023.

## Supplementary Information

**Additional file 1.**

## Data Availability

This study was submitted and analysed by the IPOP’s data protection officer (Ref. – 137/2020). The final trial dataset will only be accessed by the researchers involved in the study. Data will not be publicly available. Nonetheless, some data will be shared with other partners of the MindGAP project and might be made available to other researchers upon reasonable request to the research team.

## Abbreviations

ACTH: Adrenocorticotropic Hormone
BaSIQS: Basic Scale on Insomnia Complaints and Quality of Sleep
CA: Cancer Antigen
CCI: Client Change Interview
COPE: Committee on Publication Ethics
CORE-OM: Clinical Outcome Routine Evaluation - Outcome Measure
CNS: Central Nervous System
CRP: C-Reactive Protein
DASS-21: Depression, Anxiety Stress Scales-21
DSM: Diagnostic and Statistical Manual
DT: Distress Thermometer
ERQ: Emotion Regulation Questionnaire
EVs: Extracellular Vesicles
FCR-7: 7-item Fear of Cancer Recurrence
FFMQ: Five-Facet Mindfulness Questionnaire
FTND: Fagerström Test for Nicotine Dependence
HPLC: High-Performance Liquid Chromatography
ICD: International Classification of Diseases
IFN-γ: Interferon Gamma
IFN: Interferon
IL: Interleukin
IPAQ-SF: International Physical Activity Questionnaire - Short Form
IPOP: Instituto Português de Oncologia do Porto (Portuguese Oncology Institute of Porto)
LMM: Linear Mixed Model
MBCR: Mindfulness-Based Cancer Recovery
MBCT: Mindfulness-Based Cognitive Therapy
MBIs: Mindfulness-Based Interventions
MBSR: Mindfulness-Based Stress Reduction
NCCN: National Comprehensive Cancer Network
PSA: Prostate-Specific Antigen
PTGI: Posttraumatic Growth Inventory
RCT: Randomized Controlled Trial
SPIRIT: Standard Protocol Items: Recommendations for Intervention Trials
SSSS: Satisfaction with Social Support Scale
TAU: Treatment As Usual
TNF: Tumour Necrosis Factor
WHOQOL-Bref: World Health Organization Quality of Life – Bref
